# A cohort of GFPT1 related congenital myasthenic syndrome in China: high frequency of c.331 c > t variant

**DOI:** 10.1186/s13023-025-03823-z

**Published:** 2025-05-29

**Authors:** Jialong Zhang, Xinyu Chen, Chong Yan, Xinyu Gu, Wenhua Zhu, Xuwei Cao, Lei Zhou, Sushan Luo, Jie Lin, Zunbo Li, Jiahong Lu, Chongbo Zhao, Kai Qiao, Xuefan Yu, Jianying Xi

**Affiliations:** 1 Department of Neurology, Huashan Rare Disease Center, National Center for Neurological Disorders, Huashan Hospital, Fudan University, Shanghai, China; 2 Department of Neurology, Affiliated Hospital of Guizhou Medical University, Guiyang, 550004 China; 3 Department of Neurology, Xi’an Gaoxin Hospital, Xi’an Medical College, Xi’an, 710075 China; 4 Department of Neurology, the First Affiliated Hospital of Jilin University, Changchun, 130021 China

**Keywords:** Congenital myasthenic syndrome, Glutamine-fructose-6-phosphate transaminase 1, Tubular aggregates, Distal muscle weakness

## Abstract

**Background:**

Glutamine-fructose-6-phosphate transaminase 1 (GFPT1) is the key enzyme initiating protein *O*- and *N*-glycosylation at the postsynaptic membrane. Variants in *GFPT1* gene cause congenital myasthenic (GFPT1-CMS). However, the understanding of the phenotype and genetic spectrum of GFPT1-CMS remains limited.

**Methods:**

A total of 24 patients with GFPT1-CMS from 22 Han Chinese families across four neuromuscular disease centers were included in this study. Clinical assessments involved detailed medical histories, muscle biopsies, and electrophysiological studies. *GFPT1* variants were identified using targeted next-generation sequencing or WES. Additionally, published GFPT1-CMS case data from 2011 to 2024 were compiled and combined with this cohort for genotype-phenotype correlation analysis.

**Results:**

In addition to the limb girdle myasthenia pattern, our cohort presented with extraocular involvement including eyelid ptosis and mild ophthalmoparesis (25.0%), facial weakness (20.8%) and a relatively high prevalence of distal weakness (62.5%). Electrophysiological testing revealed myopathic changes in 95.0% of cases and decremental CMAPs in all cases during RNS. We found that c.331 C > T is a hotspot variant in GFPT1-CMS patients and may have a founder effect in the Chinese population. Patients with homozygous null variants presented a more severe phenotype, including earlier onset and more frequent bulbar involvement.

**Conclusion:**

We have described the clinical features and variant spectrum in a cohort of 24 Chinese GFPT1-CMS patients. Our findings update the understanding of clinical manifestation, pathological features and mutational spectrum in GFPT1-CMS patients.

**Supplementary Information:**

The online version contains supplementary material available at 10.1186/s13023-025-03823-z.

## Introduction

Congenital myasthenic syndrome (CMS) is a genetically and clinically heterogenous group of inherited neuromuscular junction (NMJ) disorder. Currently, more than 30 causative genes have been identified as contributing to CMS [1]. Based on the causative genes and the functions of their encoded proteins, CMS can be categorized into defects in presynaptic components, synaptic space, postsynaptic elements, and protein glycosylation [2]. Glutamine-fructose-6-phosphate transaminase 1 (GFPT1) serves as an essential enzyme that participates in the postsynaptic hexosamine biosynthetic pathway (HBP), initiating the *O*-linked and *N*-linked glycosylation of ubiquitous proteins [3]. CMS with variants in *GFPT1* was first identified in 24 patients characterized by limb-girdle myasthenia (LGM) with tubular aggregates (TAs) in 2011 [3, 4].

To date, only three large GFPT1-CMS cohorts from Germany, the United States, and French have been described [4–6], and the understanding of the phenotype and genetic spectrum of GFPT1-CMS remains limited. Here, we reported a cohort of 24 patients from 22 pedigrees in China, summarizing the clinical manifestation, pathological features, and variant spectrum of GFPT1-CMS. Additionally, We provided a summary of 108 previously reported global patients to address regional variations and genotype-phenotype correlations in GFPT1-CMS.

## Materials and methods

### Patients

Patients with confirmed diagnosis of GFPT1-CMS from four Neuromuscular disorder centers (Shanghai, Guiyang, Xi’an and Changchun) between 2008 and 2024 were included in this study. The diagnostic criteria included: (1) childhood or adult onset of symptomatic muscle weakness with or without > 10% decrement on low-frequency repetitive nerve stimulation (RNS) testing; (2) a confirmed molecular diagnosis with *GFPT1* variant identified on both alleles. This study was approved by the Institutional Review Board at Huashan Hospital, Fudan University (KY2021-753). All clinical information and biological materials used in this study were obtained with written informed consent.

### Clinical and pathological evaluation

Detailed clinical information, including age at onset, initial symptoms, family history, creatine kinase (CK) levels, electrophysiological studies, and treatment response, was retrospectively collected using a standardized questionnaire. Treatment response was assessed based on patient-reported improvement during follow-up. Muscle biopsies were performed in 13 patients. Serial frozen sections of 8 μm thickness were used for routine hematoxylin and eosin (HE), modified Gomori trichrome (MGT), nicotinamide adenine dinucleotide-tetrazolium reductase (NADH-TR) and adenosine triphosphatase (ATP) staining. Electromicroscopic examination was performed. in 4 patients (Pt 1, 9, 13 and 18).

### Molecular analyses

Genomic DNA was extracted from peripheral blood leucocytes via standard protocols. Targeted next-generation sequencing (NGS) covering 195 genes associated with hereditary myopathies and NMJ disorders (Amplicon Gene, China), including *AGRN*,* ALG2*,* ALG14*,* CHAT*,* CHRNA1*,* CHRNB1*,* CHRND*,* CHRNE*,* COLQ*,* COL13A*,* DOK7*,* DPAGT1*,* GFPT1*,* GMPPB*,* LAMB2*,* LAMA5*,* LRP4*,* MUSK*,* MYO9A*,* PREPL*,* RAPSN*,* SLC18A3*,* SLC25A1*,* SCN4A*,* SYB1*,* SYT2* and *SNAP25*) or whole exome sequence (WES) was performed in the probands. Sanger sequencing was used to confirm the variants in probands and their family members. References of nucleotides or amino acids are based upon the genomic DNA (NC_000002) and cDNA (NM_001244710.2) sequence of *GFPT1*. The pathogenicity of novel variants was interpreted following the 2015 recommendations of the American College of Medical Genetics and Genomics (ACMG) [7]. SIFT [8], PolyPhen-2 [9], and MutationTaster [10] were applied to assess the impact of the variants on protein function. To thoroughly analyze the variant profile of *GFPT1*, we extracted the *GFPT1* gene variant frequency from gnomAD (https://gnomad.broadinstitute.org/). The allele frequency of *GFPT1* in Chinese population was obtained from ChinaMAP (http://www.mbiobank.com).

### Haplotype analysis

The single-nucleotide polymorphisms (SNP) haplotype pattern was analysis in 12 patients with the most frequent variant, c.331 C> T. Four tag SNPs (rs6722492, rs6546505, rs67760762, rs6720415) were chosen according to previous studies [11, 12]. All variant descriptions were unified using HGVS sequence variant nomenclature descriptions with the Mutalyzer tool to ensure consistency and accuracy in reporting.

Data for the tag SNPs were collected from their NGS results, and for those with sequencing coverage less than 10, direct Sanger sequencing was used for genotyping. Haplotypes were reconstructed using PHASE version 2.1 [13, 14]. The control data, including 206 individuals from North China and 210 from South China, were downloaded from the 1000 Genomes Project [15].

### Literature review

The literature search was restricted to articles published in English. Two authors (JZ and XC) independently searched the PubMed (2011–2024) using the keywords “GFPT1” or “congenital myasthenic syndrome”. Cases with detailed clinical information and bi-allelic variants in *GFPT1* gene were reviewed.

### Statistical analysis

Statistical analysis was performed using GraphPad Prism 5. Quantitative variables were shown as mean ± SD. Comparison of alleles frequencies and haplotype between groups were using two-sided Fisher’s exact test/Chi-squared test. *p*<0.05 was considered to be statistically significant. Required R packages (latest version accessed on 8 March 2024) include tidyverse, dplyr, ggplot2 and circlize were used for data processing and diagram generation.

## Results

### Demographic and clinical features

Twenty-four patients with molecularly confirmed GFPT1-CMS from 22 Chinese Han pedigrees from four centers between 2008 and 2024 were included (Supplementary Table 1). Two patients (Pt 17 and 18) had been previously reported [16]. The clinical characteristics of 24 patients from 22 pedigrees are summarized in Table 1. Among 22 pedigrees, 9 (40.9%), 8 (36.4%), 3 (13.64%), 2 (9.10%) were from East, Central, West, North China, respectively. The gender ratio was 3:1 (18 males vs. 6 females), and the age of onset ranged from birth to 12 years old. Twenty-two patients (91.7%) developed muscle weakness before the age of ten years old. Six pedigrees (6/22, 27.3%) had a family history of consanguineous parents. Twenty-one out of 24 patients (87.5%) exhibited fluctuations in weakness, with 11 patients (45.8%) experiencing daily fluctuations and 18 patients (75.0%) seasonal fluctuations. Two patients (Patient 6 and Patient 11) specifically reported a worsening of weakness in hot weather. Out of 24 patients, 9 (37.5%) exhibited only proximal weakness, while 15 (62.5%) showed both proximal and distal weakness with either proximal predominance or equal involvement. Among those with distal weakness, the wrist and finger extensors were the most commonly affected. None of the patients complained of ptosis or diplopia. However, on physical examination, 6 patients (25.0%) showed ocular involvement: 5 patients (20.8%) exhibiting mild ptosis, and 3 (12.5%) showing ophthalmoplegia with very mild horizontal restriction of eye movement. No fatigue of ptosis or ophthalmoplegia was reported, either. Facial weakness was observed in 5 patients (20.8%). For bulbar weakness, three patients presented solely with dysarthria, showing no evidence of dysphagia or chewing weakness. None developed respiratory dysfunction. Two patients (8.3%) had distal joint contracture (Pt 5 − 1, 5 − 2), while 3 (12.5%) patients exhibited a high-arched palate (Pt 6, 7 and 13) and 1 patient showed scapular winging (Pt 5 − 1, 5 − 2, 6, 13).

**Table 1:** Clinical features in our cohort and global GFPT1-CMS patients

	Our cohort (*n* = 24)	Global GFPT1-CMS (*n* = 108)	*p*
**Gender**			
Female	6/24	35/99	0.470
Age at onset (y)			0.625^a^
birth 0–10 10–18 > 18	2/24 20/24 2/24 0/24	10/108 77/108 13/108 8/108	
**Fluctuation**	21/24	63/74	1^b^
**Weakness**			
Proximal Distal Neck Bulbar Ptosis Ocular Facial	24/24 15/24 20/24 3/24 5/24 3/24 5/24	108/108 42/100 36/92 16/105 10/106 4/106 14/103	- 0.109 **0.001** ^**a**^ 0.982^b^ 0.221^b^ 0.117^a^ 0.563^b^
**Elevated CK level**	10/17	41/78	0.790
**Myogenic EMG**	19/20	63/76	0.313^b^
**Positive RNS**	21/21	91/95	1^a^
**Tubular aggregates**	6/13	48/72	0.271^b^
**Benefit from treatment**			
Pyristigmine	22/22	89/91	1^a^
β2 receptor agonist	16/17	18/22	0.363^a^

y, year; EMG,electromyography; RNS, repetitive nerve stimulation; CK, creatine kinase

^a^Using Fisher exact test

^b^Using Chi-square test with continuity correction

The bold and italic values mean significant differences

Level of creatine kinase was slightly elevated in 10/17 (58.8%) patients, ranging from 220 to 557 U/L(normal range: 38–174 U/L). Nerve Conduction Velocity (NCV) was normal in most patients, but a decrement in compound muscle action potentials (CMAPs) were recorded in proximal or distal muscles in all 21 patients (100%) by repetitive nerve stimulation (RNS) at 3 Hz. Myopathic changes were identified in 19/20 (95.0%) patients by electromyogram. Muscle MRI of lower limbs was performed in 5 patients, but no remarkable changes were found. All inquired patients (22/22, 100.0%) responded well to pyridostigmine and 16/17 (94.1%) patients who were followed-up showed additional benefited from salbutamol.

### Muscle pathology

**Fig. 1:**
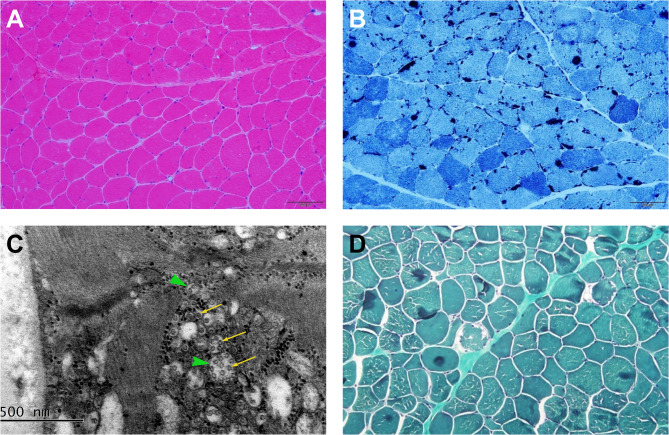
Muscle pathology of GFPT1-CMS patients. Muscle pathology showed tubular aggregates (Pt. 9) (**A**-**C**) and vacuoles (Pt. 2) (**D**)

Muscle biopsies were performed in 13 patients and 6/13 (46.2%) showed tubular aggregates (TAs) on muscle pathology (Fig. 1A, B). TAs were confirmed by election microscopy in 2 patients (Pt 9 and 18) (Fig. 1C). Rimed vacuoles (Fig. 1D) were also identified in 5/13 (38.5%) patients. One patient (1/13, 7.7%) showed Type 1 fiber predominance.

### Molecular analyses

**Fig. 2:**
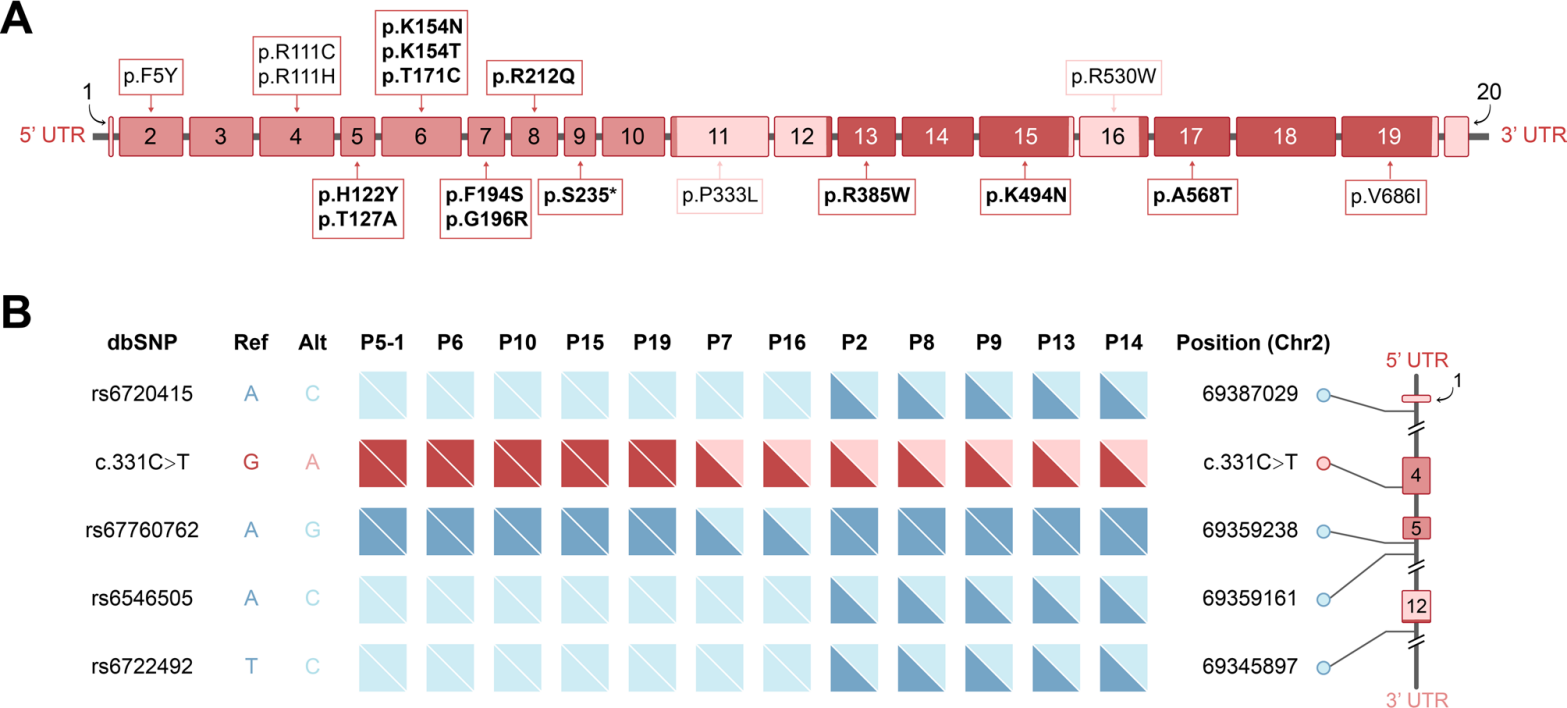
Schematic representation of *GFPT1* variants and haplotype analysis. (**A**) Schematic presentation of *GFPT1 *variants. Variants newly identified in this study are shown in bold black text. (**B**) Haplotype analysis. Red/pink triangles represent alleles with/without c. 331 C> T. Light blue triangles indicate references sequence, whereas blue triangles indicate the polymorphic variant

Half of the patients (50.0%) harbored compound heterozygous variants, while the other half carried homozygous variants. In total, 18 variants were identified in 24 patients from 22 pedigrees, including 17 missense and 1 nonsense variants (Fig. 2A). Among them, a novel nonsense, c.704 C > G (p.S235*) was classified as “pathogenic” and 11 novel missense variants as “likely pathogenic” according to the 2015 ACMG recommendation (Supplementary Table 3). The variant, c.331 C > T, was identified homozygous in 7 pedigrees and heterozygous in another 9 pedigrees in our cohort, with an allelic frequency of 52.3% (23/44), suggesting that c.331 C > T may be a hotspot variant in Chinese patients with GFPT1-CMS.

### Haplotype analysis

The data of SNP were obtained from 12 patients harboring the hotspot variant, c.331 C > T, p.R111C (Fig. 2B). All alleles (16/16) with c.331 C > T had the CGCC haplotype (Fig. 2B) and this haplotype is present in 108/416 (26.0%) alleles from normal Han Chinese population (*p* < 0.001).

### Global GFPT1-CMS

**Fig. 3:**
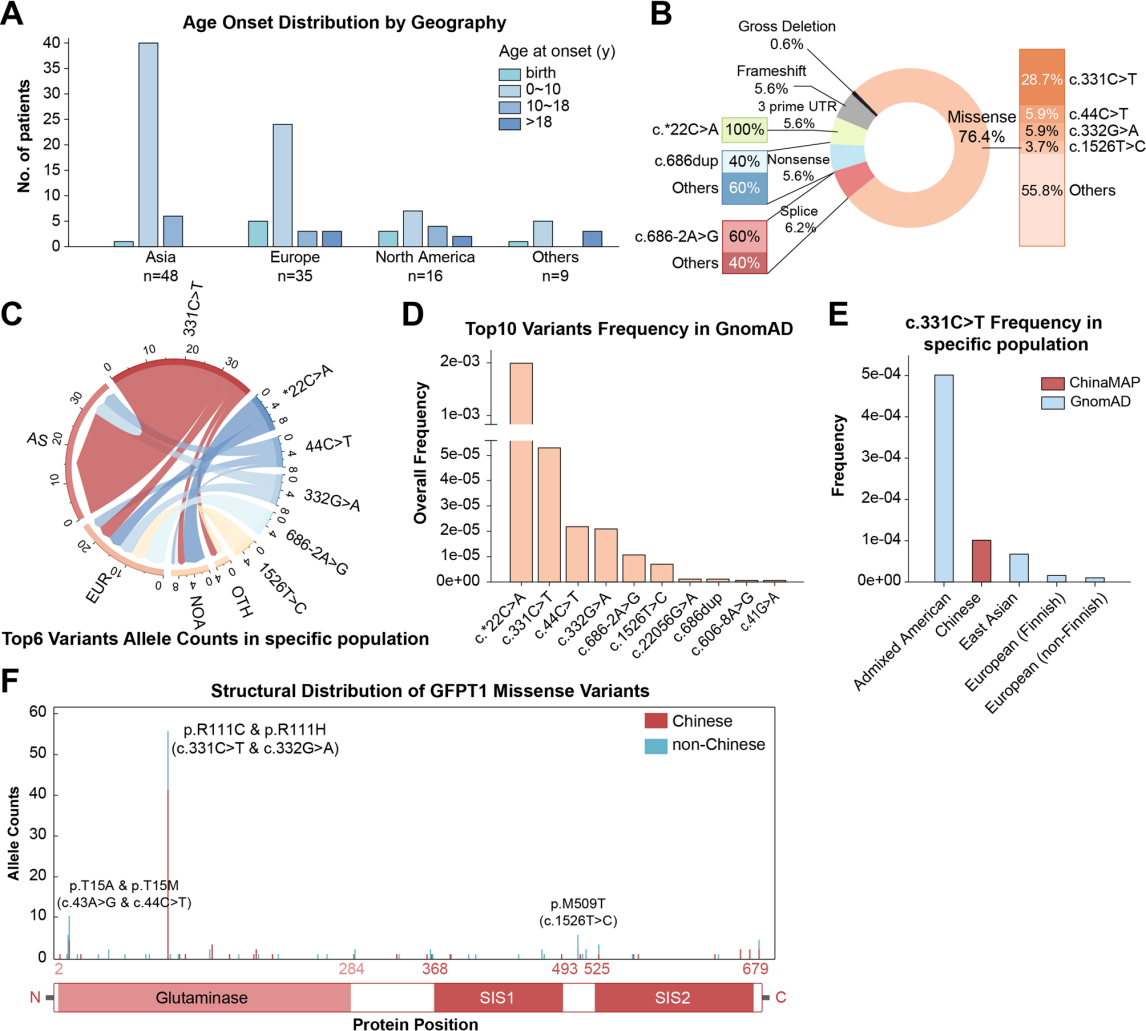
Schematic representation of the distribution and frequency analysis of *GFPT1* variants. (**A**) Age of onset distribution across different regions, categorized into birth, 0–10, 10–18, and over 18 years. (**B**) The distribution of variant types, with missense variants comprising 76.4% of the entire dataset, and within missense variants, c.331 C > T accounts for 28.7%. (**C**) Chord plot for the association between recurrent variants and ethnicity, where the thickness of the connecting bands represents the number of variants. (**D**) Allele frequency comparison of c.331 C > T between different populations. (**E**) The frequency of c.331 C > T variant between the Han Chinese and other populations. (**F**) Structural distribution of *GFPT1* variants, highlighting the allele counts in global cohort, with a high frequency of c.331 C > T variant.

Twenty-six studies fulfilling the criteria were included in this review. A total of 108 molecularly confirmed patients from 89 pedigrees were reviewed and summarized, including 24 patients from our cohort (Supplementary Tables 1 and 2). Among these patients, 48 were of Asian descent from China (38 cases), India (3 cases), Israel (2 cases), Iran (2 cases), Nepal, Japan, and South Korea (1 case each). All 35 European patients were Caucasian, hailing from Spain, Germany, Turkey, Italy, Afghanistan (Pathan), Malta, the UK, and Sweden. Sixteen patients came from North America (USA and Mexico) (Fig. 3A). The disease onset age of most patients (71.30%, 77/108) was in their childhood (between 0 and 10 years), with significant regional differences (Fig. 3A, *p* = 0.001). Additionally, compared to our cohort, the global patient population showed a lower incidence of neck muscle weakness (Table 1, *p* = 0.002).

Thirty-nine pedigrees (43.8%) carried a homozygous variant and 50 (56.2%) harbored compound heterozygous variants. Totally, 84 variants in *GFPT1* gene were identified (Supplementary Table 1), including 63 missenses, 9 frameshifts (4 deletions, 3 insertions and 2 duplications), 6 nonsenses, 4 splicing, 1 gross deletion and 1 variant in the 3’ untranslated region (c.*22C > A) (Fig. 3B).

In the global cohort, six recurrent variants were identified, with the most common being c.331 C > T, c.*22C > A, c.44 C > T, c.332G > A, c.686–2 A > G, and c.1526T > C, exhibiting allele frequencies of 21.9%, 5.6%, 4.5%, 4.5%, 3.9%, and 2.8%, respectively. Twenty-four variants were identified only in Chinese cohorts, while 47 were found only in non-Chinese cohorts. The hotspot variants vary among different ethnic groups. In Asian cohorts, the allele frequency of c.331 C > T is 34.4% (33/86), while in non-Asian cohorts, it is 14.2% (17/120) (*p* = 0.0085). The hotspot variants in Europe and North America are c.686–2 A > G and c.*22C > A, with the allele frequency of 11.5% (6/52) and 20.0% (6/30) (Fig. 3C), respectively. Notably, c.*22C > A, c.686–2 A > G, and c.1526T > C have only been detected in patients outside of Asia.

To assess whether the variant profile in the *GFPT1* gene has any ethnic predominance, we analyzed the frequency of the top ten common variants in the general population (Supplementary Table 4, Fig. 3D). c.*22C > A, which has the highest frequency, was found only in non-East Asian populations. The presence of five other common variants in the general population aligns with the data in global patients. None of the variants are unique to any specific population. We also examined differences between the Han Chinese and other populations using the ChinaMAP database. Only the c.331 C > T variant was identified in the Chinese population, with its frequency being second only to that in Admixed American (Fig. 3E).

According to the type of variants, except 13 patients with c.*22C > A, 95 patients were divided into 2 groups: patients with only missenses (group 1, *n* = 72); patients with non-missense variants, including nonsense, frameshift, splicing, gross deletion, or initiation codon variants (group 2, *n* = 23), (Table 2). Patients in group 2 showed significantly earlier onset (*p* = 0.003), higher proportion of female (*p* = 0.015), and more involvement of bulbar muscles (*p* = 0.012). No phenotype differences were found between patients with variants in the glutamine aminotransferase type 2 (GATase-2) domain and those without such variants (*p* = 0.022, Supplementary Table 5) or between patients with c.331 C > T (*n* = 28) compared to those without it (*n* = 44)(Supplementary Table 6).

**Table 2:** Comparison of clinical features between GFPT1-CMS patients with missense and non-missense variants

	With only missense variants (*n* = 72)	With non-missense variants (*n* = 23)	*p*
**Gender**			
Female	17/65	12/22	**0.015**
Age at onset (y)			
birth 0–10 10–18 > 18	4/72 56/72 7/72 5/72	6/23 6/23 1/23 0/23	**0.003** ^**a**^
Fluctuation	47/54	10/12	1.000^b^
**Weakness**			
Proximal Distal Neck Bulbar Ptosis Ocular Facial	72/72 27/69 23/62 8/71 6/72 4/72 10/71	23/23 7/18 9/18 8/21 3/21 0/21 3/19	- 0.604 0.414 **0.012** ^**b**^ 0.695^b^ 0.571^a^ 1.000
**Elevated Creatine Kinase**	27/53	9/16	0.710
**Myogenic EMG**	43/49	12/15	0.740
**Positive RNS**	65/66	15/16	0.354^a^
**Tubular aggregates**	31/43	11/19	0.270
**Benefit from treatment**			
Pyristigmine	62/63	16/17	0.382^a^
β2 receptor agonist	17/19	1/3	0.073^a^

y, year; EMG, electromyography; RNS, repetitive nerve stimulation

*p*-values were determined using Chi-square test. The bold and italic values mean significant differences

^a^Using Fisher exact test

^b^Using Chi-square test with continuity correction

## Discussion

In our study, twenty-seven cases of GFPT1-CMS accounted for 38.6% of the 70 CMS cases across four centers. In a previous study from northern China, 27.6% of 29 pedigrees were GFPT1-CMS [17]. Thus, GFPT1-CMS appears to be a common cause of CMS in the Chinese population.

The muscle weakness and reduced response to repetitive stimulation, along with the positive response to AChEI, suggest that NMJ dysfunction is central to the pathology of GFPT1-CMS. This likely results from impaired glycosylation of essential proteins, such as acetylcholine receptors (AChR), in the postsynaptic apparatus due to GFPT1 deficiency, leading to defective endplate development and impaired depolarization of the postsynaptic membrane. This phenomenon has been observed in mouse and zebrafish models [3, 18]. Immunoblot analysis confirmed that the levels of O-linked N-acetylglucosamine on muscle lysates remarkably decreased in GFPT1-CMS patients [3, 6]. Additionally, GFPT1 deficiency may have direct pathological effects on extrasynaptic regions, with tubular aggregates within muscle fibers—believed to represent misfolded proteins—being the most common finding, though the precise mechanisms are still unclear [19].

Previous studies [4–6] have shown that GFPT1-CMS presents distinct clinical features, including: (1) early onset, typically before age 10; (2) limb-girdle weakness, predominantly affecting proximal limbs; (3) absence of oculobulbar or respiratory muscle involvement; (4) a decremental response on repetitive nerve stimulation (RNS) testing; (5) pathological findings of tubular aggregates or vacuolar myopathy; and (6) a favorable response to acetylcholinesterase inhibitors (AChEI) and 3,4-diaminopyridine (3,4-DAP) [5]. Consistent with previous studies, we found all the patients showed proximal weakness. We also identified a high proportion (58.3%) of patients exhibited distal weakness, particularly in wrist or hand extension [4–6], which was not rare in the global cohort, either. Sparing of ocular weakness is considered a typical feature of GFPT1-CMS and is used to differentiate it from other CMS due to mutations in *CHRNE*, *CHRNA1* and etc [4]. However, in our cohort, 33.3% (8/24) of patients exhibited mild extraocular muscle involvement despite having no subjective complaints. Among 73 patients who underwent RNS in the global cohort, 3 patients (4.1%) showed a normal RNS result. In our experience, performing RNS in both proximal and distal muscles during symptomatic periods could increase sensitivity. However, in patients with typical symptoms but negative RNS results, SFEMG or further molecular test could also enhance the diagnostic yield. Reports on muscle imaging in GFPT1-CMS are extremely rare, with only two cases documenting signs of fatty infiltration in lower limb muscles [20]. In our cohort, no specific changes were observed on muscle imaging, suggesting a lack of correlation between the severity of muscle weakness and abnormalities seen on MRI. Muscle pathology also revealed tubular aggregates (6/13, 46.2%) and vacuoles (5/13, 38.5%), while the absence of TA cannot rule out the diagnosis of GFPT1-CMS. Regarding treatment, most patients in our cohort responded well to pyridostigmine and β2 receptor agonists, aligning with outcomes reported in global GFPT1-CMS studies.

GFPT1-related CMS also needs to be differentiated from other types of CMS, primarily including glycosylation defects (*DPAGT1*, *ALG2* and *ALG14*) and abnormalities in the AChR clustering pathway (*AGRN*, *MUSK* and *DOK7*) (see Supplementary Table 7) [21, 22]. The presence of tubular aggregates and a good response to AChEIs could help in the diagnosis. Pathogenic variants in *DPAGT1* and *ALG2* can also cause congenital disorders of glycosylation (CDG type Ij), which are associated with developmental delay and intellectual disability.

Six recurrent variants in *GFPT1* have been identified worldwide, with c.331 C > T being the most frequently reported variant, accounting for the largest number of carrier cases. Although 331 is not a mutation unique to Asian populations, the number of carrier cases in China significantly exceeds that in other countries. This regional pattern is further supported by the high frequency of c.331 C > T variant observed in our cohort, with an allelic frequency reaching 52.1% (25/48), and the 16 cases with c.331 C > T shared the same haplotype, suggesting the possibility of a founder effect in the Chinese population. Additionally, consistent with findings reported in the literature, the c. *22C > A variant in the gnomAD database, despite its highest frequency, is observed only in non-East Asian populations. These discrepancies might be explained by different genetic backgrounds.

The inclusion of past literature has expanded our GFPT1-CMS case pool to over 100 individuals, while the genotype-phenotype correlation remains unclear. Our re-analysis of 26 reported studies reveals that gender ratio, age of onset and the involvement of bulbar symptoms exhibited significant correlations with variant types (with or without null variants) among the clinical features, suggesting a more severe phenotype in patients with null variants. In addition, among cohorts with missense variants, no significant correlations were found between the hotspot variant c.331 C > T and clinical features. Therefore, based on existing records, clinical differences among GFPT1-CMS patients with different variants of the same type may not be significant. A detailed assessment of muscle strength impairment should be conducted to better distinguish their phenotypic characteristics.

A major limitation of this study was its retrospective study with relatively small sample size, which may introduce recall bias and limited the generalizability of the results. In addition, treatment responses were based on subjective reports from patients or caregivers, without quantitative outcome measures.

In conclusion, we have described the clinical features and variant spectrum in a cohort of 24 Chinese GFPT1-CMS patients from 22 pedigrees. Except for proximal muscles, extraocular, facial and distal muscle involvement could also be found in our cohort. We found a high frequency of c.331 C > T variant, which shared the same haplotype in our cohort, suggesting a possible founder effect in Chinese population. Patients with homozygous null variants of *GFPT1* experience earlier disease onset and more frequent bulbar involvement. Our findings expand the current knowledge on phenotype-genotype correlations in GFPT1-CMS.

## Electronic supplementary material

Below is the link to the electronic supplementary material.

Supplementary Material 1

## Data Availability

All data are available on reasonable request from the corresponding author.
