# Supplementary material for: A cohort of GFPT1 related congenital myasthenic syndrome in China: high frequency of c.331 c > t variant
Source: Orphanet J Rare Dis. 2025 May 29;20:259. doi: 10.1186/s13023-025-03823-z (PMC12124097; doi:10.1186/s13023-025-03823-z)
Supplement: Supplementary file 1 — Supplementary Material 1 [file 13023_2025_3823_MOESM1_ESM.docx]

**Supplementary Materials**

**A cohort of GFPT1 related Congenital Myasthenic Syndrome in China: High Frequency of c.331C>T Variant**

Jialong Zhang^1#^, Xinyu Chen^1#^, Chong Yan^1^, Xinyu Gu^1^, Wenhua Zhu^1^, Xuwei Cao^2^, Lei Zhou^1^, Sushan Luo^1^, Jie Lin^1^, Zunbo Li^3^, Jiahong Lu^1^, Chongbo Zhao^1^, Kai Qiao^1^, Xuefan Yu ^4*^ Jianying Xi^1*^

**Supplementary Table 1. Clinical Characteristics and Mutation Spectrum of GFPT1-CMS Patients in this study**

| Region | Case No. | Gender | Age at onset (y) | Fluctuation | Muscle weakness | | | | | | | CK (U/L) | EMG | RNS | Pathology | Muscle of Biopsy | Treatment | | Mutation 1 | | Mutation 2 | |
| --- | --- | --- | --- | --- | --- | --- | --- | --- | --- | --- | --- | --- | --- | --- | --- | --- | --- | --- | --- | --- | --- | --- |
|  |  |  |  |  | Proximal  MRC | Distal  MRC | Neck  MRC | Bulbar | Ptosis | Ocular  movement | Facial  MRC |  |  |  |  |  | Pyristigmine | Salbutamol | Nucleotide | Amino acid | Nucleotide | Amino acid |
| East | 1 | M | 3 | + | 3 | 4 | 2 | dysarthria | - | - | 3 | ↑ | M | + | TA | (L)vastus lateralis | NA | NA | c.1588C>T | p.R530W | c.461A>C | p.K154T |
| Central | 2 | M | 3 | + | 3 | 4 | 3 | - | - | - | 4 | - | M | + | Rimmed vacuoles | (L)biceps brachii | + | - | c.331C>T | p.R111C | c.364C>T | p.H122Y |
| Central | 3 | M | 7 | + | 4 | 5 | 4 | dysarthria | - | - | 4 | NA | M | + | NA | / | + | + | c.331C>T | p.R111C | c.331C>T | p.R111C |
| East | 4-1 | M | <10 | + | 4 | 5 | 3 | - | - | - | 5 | - | M | + | TA | (L)biceps brachii | + | + | c.586G>A | p.G196R | c.586G>A | p.G196R |
| East | 4-2 | F | <10 | + | 4 | 5 | 4 | - | - | - | 4 | - | NA | NA | NA | / | NA | NA | c.586G>A | p.G196R | c.586G>A | p.G196R |
| Central | 5-1 | M | 5 | + | 3 | 4 | 4 | dysarthria | + | + | 5 | - | M | + | NA | / | + | + | c.331C>T | p.R111C | c.331C>T | p.R111C |
| Central | 5-2 | M | 12 | + | 4 | 4 | 4 | - | - | - | 5 | NA | M | + | NA | / | + | + | c.331C>T | p.R111C | c.331C>T | p.R111C |
| East | 6 | M | 7 | + | 4 | 4 | 4 | - | + | + | 5 | ↑ | M | + | Rimmed vacuoles | (R)biceps brachii | + | + | c.331C>T | p.R111C | c.331C>T | p.R111C |
| East | 7 | F | 2 | + | 3 | 4 | 3 | - | - | - | 5 | NA | M | + | NA | / | + | + | c.331C>T | p.R111C | c.704C>G | p.S235* |
| West | 8 | M | 2 | - | 3 | 5 | 4 | - | - | - | 5 | NA | NA | + | Rimmed vacuoles | (L)biceps brachii | + | + | c.331C>T | p.R111C | c.1702G>A | p.A568T |
| Central | 9 | M | 6 | + | 3 | 5 | 3 | - | - | - | 5 | ↑ | M | + | TA | (L)deltoid | + | + | c.331C>T | p.R111C | c.332G>A | p.R111H |
| East | 10 | M | 5 | + | 3 | 4 | 4 | - | - | - | 5 | ↑ | M | + | - | (L)biceps brachii | + | + | c.331C>T | p.R111C | c.331C>T | p.R111C |
| East | 11 | M | 10 | + | 3 | 5 | 3 | - | + | - | 4 | - | M | + | - | (L)biceps brachii | + | + | c.379A>G | p.T127A | c.462G>C | p.K154N |
| East | 12 | M | <10 | + | 4 | 4 | 2 | - | - | - | 5 | ↑ | M | + | NA | / | + | NA | c.332G>A | p.R111H | c.332G>A | p.R111H |
| East | 13 | F | 2 | - | 3 | 5 | 1 | - | - | - | 5 | - | M | + | Type 1 fiber predominance | (L)biceps brachii | + | + | c.331C>T | p.R111C | c.1153C>T | p.R385W |
| East | 14 | M | 12 | + | 3 | 3 | 3 | - | - | + | 5 | ↑ | M | + | NA | / | + | + | c.331C>T | p.R111C | c.511_512delinsTG | p.T171C |
| Central | 15 | M | birth | + | 3 | 4 | 5 | - | - | - | 5 | NA | M | + | NA | / | + | + | c.331C>T | p.R111C | c.331C>T | p.R111C |
| North | 16 | M | 4 | + | 4 | 4 | 4 | - | + | - | 5 | ↑ | M | + | Rimmed vacuoles+Varied fiber sizes | (L)biceps brachii | + | NA | c.331C>T | p.R111C | c.1482G>C | p.K494N |
| North | 17 | M | 7 | - | 3 | 4 | 5 | - | - | - | 5 | ↑ | M | NA | TA | (L)biceps brachii | + | NA | c.331C>T | p.R111C | c.635G>A | p.R212Q |
| West | 18 | F | 4 | + | 4 | 4 | 5 | - | + | - | 5 | ↑ | M | + | TA+Rimmed vacuoles | (L)biceps brachii | + | + | c.14T>A | p.F5Y | c.581T>C | p.F194S |
| Central | 19 | M | 4 | + | 3 | 5 | 4 | - | - | - | 5 | ↑ | NA | + | TA | (L)biceps brachii | + | NA | c.331C>T | p.R111C | c.331C>T | p.R111C |
| West | 20 | M | 7 | + | 4 | 5 | 5 | - | - | - | 5 | NA | NA | NA | NA | / | + | + | c.331C>T | p.R111C | c.331C>T | p.R111C |
| Central | 21 | F | 18m | + | 3 | 4 | 4 | - | - | - | 5 | NA | - | + | NA | / | + | + | c.331C>T | p.R111C | c.998C>T | p.P333L |
| Central | 22 | F | 3 | + | 4 | 4 | 5 | - | - | - | 5 | - | M | + | NA | / | + | NA | c.2056G>A | p.V686I | c.2056G>A | p.V686I |

MRC, Medical Research Council grade; TA, Tubular aggregates; NA, Not Available

**Supplementary Table 2. Summary of Clinical Features and Mutation Spectrum in Previously Reported GFPT1-CMS Patients**

| Origin | Case No. | Gender | Age at onset (y) | Fluctuation | Muscle weakness | | | | | | | CKb(U/L) | EMG | RNS | Pathology | Treatment | | Mutation 1 | | Mutation 2 | | Reference |
| --- | --- | --- | --- | --- | --- | --- | --- | --- | --- | --- | --- | --- | --- | --- | --- | --- | --- | --- | --- | --- | --- | --- |
|  |  |  |  |  | Proximal | Distal | Neck | Bulbar | Ptosis | Opthal-moparesis | Facial |  |  |  |  | Pyristigmine | Salbutamol | Nucleotide | Amino acid | Nucleotide | Amino acid |  |
| China | 23-1 | F | 7 | + | + | - | - | - | - | - | - | ↑ | NA | + | TA | + | NA | c.462G>C | p.K154N | c.1088A>G | p.N363S | Luo, et al. 2019. |
| China | 23-2 | M | 5 | + | + | - | - | - | - | - | - | - | NA | + | NA | + | NA | c.462G>C | p.K154N | c.1088A>G | p.N363S | Luo, et al. 2019. |
| China | 24 | M | 6 | + | + | - | - | - | - | - | - | ↑ | M | + | TA | + | NA | c.1100A>G | p.Y367C | c.1690G>T | p.G564C | Zhao, et al. 2021 |
| China | 25 | M | 3 | + | + | - | - | - | - | - | - | - | M | + | Type 1 fiber predominance | + | NA | c.76G>A | p.G26S | c.871G>A | p.V291I |  |
| China | 26 | F | 2 | NA | + | - | - | - | - | - | - | NA | M | NA | NA | NA | NA | c.331C>T | p.R111C | c.331C>T | p.R111C | Zhao, et al. 2021 |
| China | 27 | M | 6 | NA | + | - | - | - | - | - | - | ↑ | NA | + | TA | + | NA | c.2003T>C | p.V668A | c.2003T>C | p.V668A |  |
| China | 28 | M | 5 | NA | + | - | - | - | - | - | - | ↑ | M | + | TA | + | NA | c.44C>T | p.T15M | c.44C>T | p.T15M |  |
| China | 29 | F | 17 | NA | + | - | + | + | - | - | - | - | M | + | TA | + | NA | c.736A>T | p.R246* | c.1927A>C | p.T643P |  |
| China | 30 | M | 7 | NA | + | - | - | - | - | - | - | - | NA | + | TA | + | NA | c.2029C>T | p.H677T | c.2029C>T | p.H677T |  |
| China | 31 | M | 5 | + | + | + | - | - | - | - | - | - | - | + | Rimmed vacuoles | + | + | c.331C>T | p.R111C | c.332G>A | p.R111H | Jiang, et al. 2021 |
| China | 32 | M | 7 | - | + | + | - | - | - | - | - | - | M | + | TA | + | + | c.331C>T | p.R111C | c.1534C>T | p.R512W |  |
| China | 33 | M | 13 | + | + | - | - | - | - | - | - | ↑ | M | + | TA | + | NA | c.331C>T | p.R111C | c.331C>T | p.R111C | An, et al 2022 |
| China | 34 | M | 9 | + | + | - | - | - | - | - | - | - | M | + | TA | + | NA | c.331C>T | p.R111C | c.331C>T | p.R111C |  |
| China | 35 | F | 6 | + | + | - | - | - | - | - | - | - | M | + | TA | + | NA | c.44C>T | p.T15M | c.44C>T | p.T15M |  |
| Korean | 36 | M | 13 | - | + | - | - | - | - | - | - | ↑ | M | + | TA | + | NA | c.766G>C | p.E256Q | [c.1550T>C](https://test.mutalyzer.nl/name-checker?description=NM_001244710.1%3Ac.1550T%3EC) | p.M517T | Huh, et al. 2012. |
| Iran | 37-1 | M | 6 | + | + | + | NA | - | - | - | + | ↑ | M | + | TA | + | NA | c.1096G>T | p.D366Y | c.1096G>T | p.D366Y | Guergueltcheva, et al. 2012. |
| Iran | 37-2 | M | 6 | + | + | + | NA | - | - | - | + | ↑ | M | + | NA | + | NA | c.1096G>T | p.D366Y | c.1096G>T | p.D366Y |  |
| Turkey | 38 | F | 6 | + | + | - | - | - | - | - | - | - | M | + | TA | + | NA | c.719G>A | p.W240* | c.719G>A | p.W240* |  |
| Libya | 39-1 | M | 6 | + | + | - | - | - | - | - | - | - | M | + | TA | + | NA | c.331C>T | p.R111C | c.331C>T | p.R111C |  |
| Libya | 39-2 | M | 6 | + | + | - | - | - | - | - | - | - | M | + | NA | + | NA | c.331C>T | p.R111C | c.331C>T | p.R111C |  |
| Libya | 39-3 | F | 6 | + | + | - | - | - | - | - | - | - | M | + | NA | + | NA | c.331C>T | p.R111C | c.331C>T | p.R111C |  |
| Libya | 39-4 | F | 6 | + | + | - | - | + | - | - | - | - | M | + | NA | + | NA | c.331C>T | p.R111C | c.331C>T | p.R111C |  |
| Libya | 39-5 | F | 6 | + | + | - | - | - | - | - | - | - | M | + | NA | + | NA | c.331C>T | p.R111C | c.331C>T | p.R111C |  |
| Spain | 40-1 | M | 14 | - | + | + | - | - | - | - | - | ↑ | M | + | TA | + | NA | c.1529T>C | p.M510T | c.*22C>A |  |  |
| Spain | 40-2 | F | 40 | - | + | - | + | - | - | - | - | - | M | + | NA | NA | NA | c.1529T>C | p.M510T | c.*22C>A |  |  |
| Spain | 40-3 | M | 10 | + | + | + | - | - | - | - | - | ↑ | M | + | TA | + | NA | c.1529T>C | p.M510T | c.*22C>A |  |  |
| Germany | 41 | M | 5 | + | + | - | - | - | - | - | - | ↑ | NA | + | TA | + | NA | c.362T>C | p.I121T | c.128A>T | p.D43V |  |
| UK | 42 | M | 8 | - | + | + | + | - | - | - | + | ↑ | M | + | TA | + | NA | c.1208G>A | p.R403H | c.1355G>A | p.R452H |  |
| UK | 43 | M | 6 | + | + | + | - | - | - | - | + | ↑ | M | + | TA | + | NA | c.44C>T | p.T15M | c.1540C>T | p.R514W |  |
| Germany | 44 | F | 13 | + | + | - | - | - | - | - | - | - | M | + | TA | + | NA | c.*22C>A |  | c.595G>T | p.V199F |  |
| Spain | 45 | F | 1 | + | + | - | - | - | - | - | - | - | M | + | TA | + | NA | c.1588C>T | p.R530W | c.1588C>T | p.R530W |  |
| Spain | 46 | M | 7 | - | + | - | - | - | - | - | - | - | M | - | Inclusion | + | NA | c.1526T>C | p.M509T | c.1526T>C | p.M509T |  |
| Spain | 47-1 | F | 1 | + | + | + | + | - | - | - | - | - | M | + | NA | + | NA | c.*22C>A |  | c.1332_1335dup | p.D446Sfs*11 | |
| Spain | 47-2 | M | 5 | + | + | - | + | - | - | - | - | - | M | + | TA | NA | NA | c.*22C>A |  | c.1332_1335dup | p.D446Sfs*11 | |
| Sweeden | 48 | M | <10 | + | + | - | - | - | - | - | - | ↑ | M | + | TA | + | NA | c.331C>T | p.R111C | c.222_223insA | p.Q76Afs*11 | |
| Italy | 49-1 | M | 10 | - | + | - | - | - | - | - | - | ↑ | M | + | TA | + | NA | c.43A>G | p.T15A | c.621_622del | p.L208Dfs*6 | |
| Italy | 49-2 | M | 7 | - | + | - | - | - | - | - | - | ↑ | M | + | TA | + | NA | c.43A>G | p.T15A | c.621_622del | p.L208Dfs*6 | |
| Malta | 50-1 | F | 8 | + | + | NA | NA | NA | NA | NA | NA | ↑ | NA | - | - | + | NA | c.1526T>C | p.M509T | c.768_769insA | p.S257Ifs*24 | |
| Malta | 50-2 | M | 7 | + | + | NA | NA | NA | NA | NA | NA | ↑ | NA | NA | - | + | NA | c.1526T>C | p.M509T | c.768_769insA | p.S257Ifs*24 | |
| Mayo | 51 | M | 1 | NA | + | + | - | - | - | - | - | NA | - | + | TA | + | NA | c.1754_1770dup17 | p.G591Lfs*9 | c.*22C>A |  | Selcen, et al. 2013. |
| Mayo | 52 | F | 8 | NA | + | + | - | - | - | - | - | NA | - | + | TA+Type 1 fiber preponderance | + | NA | c.1688G>C | p.R563P | c.*22C>A |  |  |
| Mayo | 53 | F | 12 | NA | + | + | - | - | - | - | - | NA | - | + | Type 1 fiber predominance | + | NA | c.606-8A>G | | c.*22C>A |  |  |
| Mayo | 54 | M | 19 | NA | + | + | - | - | - | - | - | NA | - | + | TA+Rimmed vacuoles | + | NA | c.338A>G | p.D113G | c.1529T>C | p.M510T |  |
| Mayo | 55 | M | 12 | NA | + | + | - | - | - | - | - | NA | - | + | - | + | NA | c.49C>T | p.R17* | c.*22C>A |  |  |
| Mayo | 56 | F | birth | NA | + | + | + | + | - | - | - | NA | - | + | TA+Type 1 fiber preponderance+Rimmed vacuoles | - | NA | c.686-2A>G | splicing | c.964C>T | p.R322* |  |
| Mayo | 57 | M | 10 | NA | + | + | - | - | - | - | - | ↑ | - | + | TA | + | NA | c.331C>T | p.R111C | c.331C>T | p.R111C |  |
| Mayo | 58 | F | 9 | NA | + | + | - | - | - | - | - | NA | - | + | TA+Rimmed vacuoles | + | NA | c.1103C>T | p.T368I | c.1391delA | p.I464Tfs*41 | |
| Mayo | 59 | M | 4 | NA | + | + | - | - | - | - | - | NA | - | + | TA | + | NA | c.1_2del2 | p.M1fs*2 | c.44C>T | p.T15M |  |
| Mayo | 60 | M | 3 | NA | + | + | - | - | - | - | - | NA | - | + | NA | + | NA | c.2056G>A | p.V686I | c.2056G>A | p.V686I |  |
| Mayo | 61 | M | 15 | NA | + | + | - | - | - | - | - | NA | M | + | NA | + | NA | c.875T>C | p.I292T | c.875T>C | p.I292T |  |
| USA | 62 | F | 13 | + | + | + | + | - | - | - | + | ↑ | M | - | Type 1 fiber predominance | + | NA | c.606-8A>G | splicing | c.*22C>A |  | Maselli, et al. 2013. |
| Israel | 63 | NA | 5 | NA | + | NA | NA | NA | - | - | NA | NA | NA | NA | NA | NA | NA | c.35T>C | p.V12A | c.1157G>A | p.R386H | Aharoni, et al. 2017. |
| Caucasian | 64 | NA | <10 | + | + | NA | NA | - | - | - | - | ↑ | NA | + | TA | + | NA | c.207G＞A | p.L69L | c.332G>A | p.R111H | Bauche, et al. 2017. |
| Caucasian | 65 | F | birth | + | + | - | + | + | - | - | - | ↑ | M | + | TA | + | NA | c.331C>T | p.R111C | c.331C>T | p.R111C |  |
| Caucasian | 66 | NA | 6 | + | + | - | NA | - | - | - | - | ↑ | NA | + | TA | + | NA | c.1228A>C | p.T410P | c.1550T>G | p.M517R |  |
| Caucasian | 67-1 | F | 6 | + | + | - | NA | - | + | - | - | - | NA | + | TA | + | NA | c.331C>T | p.R111C | c.2056-1G>C | splicing |  |
| Caucasian | 67-2 | F | 2.5 | + | + | - | NA | - | - | - | - | - | NA | + | TA | + | NA | c.331C>T | p.R111C | c.2056-1G>C | splicing |  |
| Caucasian | 68 | NA | 24 | + | + | - | NA | - | + | - | - | ↑ | NA | + | NA | + | NA | c.332G>A | p.R111H | c.*22C>A |  |  |
| Caucasian | 69 | NA | 15 | + | + | + | NA | - | - | - | - | ↑ | NA | + | TA | + | NA | c.332G>A | p.R111H | c.1003A>T | p.M335L |  |
| Caucasian | 70-1 | NA | 22 | + | + | - | NA | - | - | - | - | - | NA | + | NA | + | NA | c.44C>T | p.T15M | c.44C>T | p.T15M |  |
| Caucasian | 70-2 | NA | <10 | + | + | - | NA | - | - | - | - | - | NA | + | NA | + | NA | c.44C>T | p.T15M | c.44C>T | p.T15M |  |
| Caucasian | 71 | NA | <10 | + | + | + | NA | - | + | + | - | ↑ | NA | + | NA | NA | NA | c.332G>A | p.R111H | c.793C>G | p.L265V |  |
| Caucasian | 72 | NA | <10 | + | + | - | NA | - | + | - | + | ↑ | NA | + | NA | NA | NA | c.1936A>G | p.I646V | c.1010-50_1105+51del | p.G337T3 68del | |
| Turkey | 73 | M | 13m | NA | + | + | + | - | - | - | - | ↑ | M | NA | Dystrophic pattern  findings | NA | NA | c.686-2A>G |  | c.686-2A>G |  | Uluç Yiş, et al. 2017 |
| Spain | 74 | F | birth | + | + | + | + | - | - | - | - | NA | NA | NA | NA | + | NA | c.686-2A>G |  | c.686-2A>G |  | D. Natera-de Benito, et al. 2017 |
| Indian | 75 | M | 11 | NA | + | - | - | - | - | - | - | NA | NA | + | NA | + | - | c.158A>G | p.N53S | c.158A>G | p.N53S | Selvam, et al. 2018. |
| Indian | 76 | M | 9 | NA | + | - | - | - | - | - | - | NA | NA | + | NA | NA | NA | c.1421G>A | p.R474Q | c.1422G>C | p.R474R |  |
| Indian | 77 | F | 1.5 | NA | + | - | - | - | - | - | - | NA | NA | + | NA | NA | NA | c.540A>G | p.Q180Q | c.266T>C | p.L89P |  |
| Japan | 78 | F | 8 | + | + | - | + | + | - | - | + | - | M | + | TA | + | - | c.776_777insG | p.D260Gfs*21 | c.776_777insG | p.D260Gfs*21 | Matsumoto, et al. 2018. |
| Nepali | 79-1 | M | 5 | NA | + | - | - | - | - | - | - | - | M | + | RRF | + | NA | c.41G>T | p.R14L | c.41G>T | p.R14L | Helman, et al. 2019. |
| Nepali | 79-2 | M | 1.5 | NA | + | - | - | - | - | - | - | - | NA | + | NA | + | NA | c.41G>T | p.R14L | c.41G>T | p.R14L |  |
| Afghanistani Pathan | 80-1 | M | birth | NA | + | + | + | + | - | - | - | NA | NA | NA | RRF | NA | NA | c.452C>A | p.T151K | c.452C>A | p.T151K |  |
| Afghanistani Pathan | 80-2 | M | birth | NA | + | + | + | + | - | - | - | NA | NA | NA | NA | NA | NA | c.452C>A | p.T151K | c.452C>A | p.T151K |  |
| Hispanic | 81 | F | birth | NA | + | NA | + | + | - | - | + | NA | M | + | NA | + | NA | c.686dupC | p.R230* | c.686dupC | p.R230* | Szelinger, et al. 2020. |
| Mexican | 82-1 | M | birth | NA | + | NA | + | + | - | - | NA | - | NA | + | - | NA | - | c.686dupC | p.R230* | c.686dupC | p.R230* |  |
| Mexican | 82-2 | M | birth | NA | + | NA | + | + | + | - | NA | NA | NA | NA | Rimmed vacuoles | + | NA | c.686dupC | p.R230* | c.686dupC | p.R230* |  |
| USA | 83 | M | 8 | NA | + | NA | NA | + | - | - | + | NA | NA | + | NA | + | NA | c.41G>A | p.R14Q | c.41G>A | p.R14Q | Prior DE, et al 2021 |
| Brazil | 84 | F | 23 | + | + | - | - | - | - | - | - | NA | M | + | TA | + | NA | c.652T>G | p.S218P | c.275C>T | p.A92V | Eduardo P. Estephan, et al 2021 |
| Brazil | 85 | F | 33 | + | + | - | - | - | - | - | - | NA | M | + | TA | + | NA | c.1429G>A | p.D477N | c.67C>G | p.L23V |  |
| Germany | 86-1 | M | 18m | NA | + | - | - | + | - | - | - | ↑ | M | NA | Rimmed vacuoles | NA | NA | c.41G>A | p.R14Q | c.1265_1268del | p.F422Wfs*26 | Alexander Mensch, et al 2022 |
| Germany | 86-2 | F | birth | NA | + | - | - | + | - | - | - | - | M | NA | Rimmed vacuoles | NA | NA | c.41G>A | p.R14Q | c.1265_1268del | p.F422Wfs*26 |  |
| USA | 87 | M | 50 | NA | + | + | - | - | - | - | - | ↑ | M | + | - | + | NA | c.7+2T>G | | c.*22C>A |  | Erika K, et al 2023 |
| Turkey | 88 | M | 10 | NA | + | - | - | - | - | - | - | ↑ | M | NA | Dystrophic pattern  findings | NA | NA | c.686-2A>G | | c.686-2A>G | | Özlem Özsoy, et al 2023 |
| Australia | 89 | F | 69 | - | + | - | - | - | - | - | - | - | - | + | TA | - | NA | c.1526T>C | p.M509T | c.1526T>C | p.M509T | Shadi El-Wahsh, et al 2023 |

TA, Tubular aggregates; NA, Not Available

**Supplementary Table 3. Novel GFPT1 variants identified in this study**

| **Type** | **Exon** | **NM_001244710** | **NP_001231639** | **ACMG** | **PVS1** | **PS1** | **PS2** | **PS3** | **PS4** | **PM1** | **PM2** | **PM3** | **PM4** | **PM5** | **PM6** | **PP1** | **PP2** | **PP3** | **PP4** | **PP5** | **SIFT**  **score** | **SIFT**  **pred** | **Polyphen2 HVAR score** | **Polyphen2 HVAR pred** | **Mutation Taster score** | **Mutation Taster pred** | **ESP6500 ALL MAF** | **1000Genome ALL MAF** | **ExAC ALL MAF** |
| --- | --- | --- | --- | --- | --- | --- | --- | --- | --- | --- | --- | --- | --- | --- | --- | --- | --- | --- | --- | --- | --- | --- | --- | --- | --- | --- | --- | --- | --- |
| Missense | Ex2 | c.14T>A | p.F5Y | LP | 0 | 0 | 0 | 0 | 0 | 1 | 1 | 0 | 0 | 0 | 0 | 1 | 1 | 1 | 0 | 0 | 0.003 | D | 0.998 | D | 1 | D | 0 | 0 | 0 |
|  | Ex5 | c.364C>T | p.H122Y | LP | 0 | 0 | 0 | 0 | 0 | 1 | 1 | 0 | 0 | 0 | 0 | 1 | 1 | 1 | 0 | 0 | 0 | D | 0.917 | D | 1 | D | 0 | 0 | 0 |
|  | Ex5 | c.379A>G | p.T127A | LP | 0 | 0 | 0 | 0 | 0 | 1 | 1 | 0 | 0 | 0 | 0 | 1 | 1 | 1 | 0 | 0 | 0.013 | D | 0.998 | D | 1 | D | 0 | 0 | 0 |
|  | Ex6 | c.461A>C | p.K154T | LP | 0 | 0 | 0 | 0 | 0 | 1 | 1 | 0 | 0 | 0 | 0 | 0 | 1 | 1 | 0 | 0 | 0.005 | D | 0.999 | D | 1 | D | 0 | 0 | 0 |
|  | Ex6 | c.462G>C | p.K154N | LP | 0 | 0 | 0 | 0 | 0 | 1 | 1 | 0 | 0 | 0 | 0 | 1 | 1 | 1 | 0 | 0 | 0.003 | D | 0.999 | D | 1 | D | 0 | 0 | 0 |
|  | Ex6 | c.511_512delinsTG | p.T171C | LP | 0 | 0 | 0 | 0 | 0 | 1 | 1 | 0 | 0 | 0 | 0 | 1 | 1 | 1 | 0 | 0 | 0 | D | 0.5 | D | 0.984 | D | 0 | 0 | 0 |
|  | Ex7 | c.581T>C | p.F194S | LP | 0 | 0 | 0 | 0 | 0 | 1 | 1 | 0 | 0 | 0 | 0 | 1 | 1 | 1 | 0 | 0 | 0.001 | D | 0.268 | B | 0.99984 | D | 0 | 0 | 0 |
|  | Ex8 | c.635G>A | p.R212Q | LP | 0 | 0 | 0 | 0 | 0 | 1 | 1 | 0 | 0 | 0 | 0 | 1 | 1 | 1 | 0 | 0 | 0.035 | D | 0.49 | P | 0.99999 | D | 0 | 0 | 0 |
|  | Ex13 | c.1153C>T | p.R385W | LP | 0 | 0 | 0 | 0 | 0 | 1 | 1 | 0 | 0 | 0 | 0 | 1 | 1 | 1 | 0 | 0 | 0 | D | 0.991 | D | 1 | D | 0 | 0 | 0 |
|  | Ex15 | c.1482G>C | p.K494N | LP | 0 | 0 | 0 | 0 | 0 | 1 | 1 | 0 | 0 | 0 | 0 | 1 | 1 | 1 | 0 | 0 | 0 | D | 0.957 | D | 1 | D | 0 | 0 | 0 |
|  | Ex17 | c.1702G>A | p.A568T | LP | 0 | 0 | 0 | 0 | 0 | 1 | 1 | 0 | 0 | 0 | 0 | 1 | 1 | 1 | 0 | 0 | 0.006 | D | 0.07 | B | 1 | D | 0 | 0 | 0 |
| Nonsense | Ex9 | c.704C>G | p.S235* | P | 1 | 0 | 0 | 0 | 0 | 0 | 1 | 0 | 1 | 0 | 0 | 1 | 0 | 0 | 0 | 0 | - | - | - | - | 1 | A | 0 | 0 | 0 |

Ex: Exon; LP: likely pathogenic; P: pathogenic

**Supplementary Table 4. Top 10 variants frequency in gnomAD**

| **Genetic Ancestry Group** | **c.*22C>A** | **c.331C>T** | **c.44C>T** | **c.332G>A** | **c.686-2A>G** | **c.1526T>C** | **c.2056G>A** | **c.686dup** | **c.41G>A** | **c.606-8A>G** |
| --- | --- | --- | --- | --- | --- | --- | --- | --- | --- | --- |
| Admixed American | 1.49E-03 | 1.00E-03 | 1.67E-05 | 0.00E+00 | 1.87E-04 | 0.00E+00 | 0.00E+00 | 0.00E+00 | 0 | 0.00E+00 |
| African/African American | 4.13E-04 | 0.00E+00 | 2.69E-05 | 1.33E-05 | 0.00E+00 | 0.00E+00 | 0.00E+00 | 3.39E-05 | 0 | 0.00E+00 |
| Ashkenazi Jewish | 1.04E-04 | 0.00E+00 | 0.00E+00 | 0.00E+00 | 0.00E+00 | 0.00E+00 | 0.00E+00 | 0.00E+00 | 0 | 0.00E+00 |
| East Asian | 0.00E+00 | 1.34E-04 | 0.00E+00 | 4.46E-05 | 0.00E+00 | 0.00E+00 | 0.00E+00 | 0.00E+00 | 0 | 0.00E+00 |
| European (non-Finnish) | 2.50E-03 | 1.87E-05 | 2.67E-05 | 2.54E-05 | 1.70E-06 | 9.33E-06 | 8.71E-07 | 0.00E+00 | 0 | 8.69E-07 |
| European (Finnish) | 3.18E-04 | 3.13E-05 | 0.00E+00 | 0.00E+00 | 0.00E+00 | 0.00E+00 | 1.74E-05 | 0.00E+00 | 1.573E-05 | 0.00E+00 |
| Middle Eastern | 0.00E+00 | 0.00E+00 | 0.00E+00 | 0.00E+00 | 1.65E-04 | 1.65E-04 | 0.00E+00 | 0.00E+00 | 0 | 0.00E+00 |
| South Asian | 1.13E-05 | 0.00E+00 | 1.11E-05 | 2.20E-05 | 0.00E+00 | 0.00E+00 | 0.00E+00 | 0.00E+00 | 0 | 0.00E+00 |
| Remaining | 1.97E-03 | 1.60E-05 | 3.24E-05 | 1.60E-05 | 6.44E-05 | 0.00E+00 | 0.00E+00 | 0.00E+00 | 0 | 0.00E+00 |
| Total | 1.97E-03 | 5.64E-05 | 2.32E-05 | 2.23E-05 | 1.13E-05 | 7.44E-06 | 1.30E-06 | 1.26E-06 | 6.278E-07 | 6.32E-07 |

**Supplementary Table 5. Comparison between GFPT1-CMS patients based on domains of GFPT1 variants**

| Item | G/G  (n=51) | G/Non-G  (n=21) | Non-G/Non-G  (n=15) | p |
| --- | --- | --- | --- | --- |
| Gender  Female | 13/47 | 7/19 | 4/14 | 0.747 |
| Age at onset (y) |  |  |  |  |
| birth  0-10  10-18  >18 | 7/51  38/51  4/51  2/51 | 1/21  15/21  3/21  2/21 | 0/15  13/15  1/15  1/15 | 0.574 |
| Fluctuation | 35/37 | 11/15 | 6/9 | **0.022**^a, b^ |
| Weakness  Proximal  Distal  Neck  Bulbar  Ptosis  Ocular  Facial | 51/51  16/46  20/46  10/51  6/51  4/51  6/49 | 21/21  7/18  6/17  4/18  1/19  0/19  2/18 | 15/15  7/15  2/12  0/15  0/15  0/15  3/15 | -  0.694  0.227  0.153  0.476  0.471  0.667 |
| Elevated Creatine Kinase | 18/37 | 9/15 | 6/11 | 0.829 |
| Myogenic EMG | 33/35 | 11/14 | 8/11 | 0.082 |
| Positive RNS | 45/45 | 16/17 | 14/15 | 0.234 |
| Tubular aggragates | 19/28 | 10/18 | 9/10 | 0.208 |
| Benefit from treatment  Pyristigmine | 44/44 | 17/17 | 13/14 | 0.187 |
| β2 receptor agonist | 14/17 | 4/4 | 0/0 | 0.511 |

y, year; EMG, electromyography; RNS, repetitive nerve stimulation; CK, creatine kinase

G/G: Patients with both alleles having variants in the glutamine amidotransferase type 2 domain.

G/Non-G: Patients with one allele having a variant in the glutamine amidotransferase type 2 domain.

Non-G/Non-G: Patients with neither allele having variants in the glutamine amidotransferase type 2 domain.

p-values were determined using Fisher’s exact test. The bold and italic values mean significant differences

a"G/G" vs "G/Non-G"

b"G/G" vs "Non-G/Non-G"

**Supplementary Table 6. Comparison between GFPT1-CMS patients with and without c.331C>T variant**

| Item | R111C (n=28) | Non-R111C(n=44) | p |
| --- | --- | --- | --- |
| Gender  Female | 7/28 | 10/37 | 0.854 |
| Age at onset (y) |  |  |  |
| birth  0-10  10-18  >18 | 2/28  23/28  3/28  0/28 | 2/44  33/44  4/44  5/44 | 0.347^a^ |
| Fluctuation | 22/25 | 25/29 | 1.000^a^ |
| Weakness  Proximal  Distal  Neck  Bulbar  Ptosis  Ocular  Facial | 28/28  13/28  14/28  4/28  3/28  3/28  2/28 | 44/44  14/41  9/34  4/43  3/44  1/44  8/43 | -  0.305  0.056  0.704  0.672  0.292  0.314^b^ |
| Elevated Creatine Kinase | 10/21 | 17/32 | 0.695 |
| Myogenic EMG | 22/25 | 21/24 | 1.000^a^ |
| Positive RNS | 26/26 | 39/40 | 1.000^a^ |
| Tubular aggragates | 9/16 | 22/27 | 0.152^b^ |
| Benefit from treatment  Pyristigmine | 27/27 | 35/36 | 1.000^a^ |
| β2 receptor agonist | 7/7 | 3/4 | 0.386^a^ |

y, year; EMG, electromyography; RNS, repetitive nerve stimulation

p-values were determined using Chi-square test.

^a^Using Fisher exact test.

^b^Using Chi-square test with continuity correction

**Supplementary Table 7. Differential Diagnosis of Limb Girdle-CMS**

| Gene | Onset age | Additional clinical feature | CNS involvement | CK level | Low frequency RNS | Muscle  pathology | Treatment response | | |
| --- | --- | --- | --- | --- | --- | --- | --- | --- | --- |
|  |  |  |  |  |  |  | Pyristigmine | Salbutamol | Amifampridine |
| *GFPT1* | childhood to adulthood | / | no | normal; occasionally high | decrement | TA | effective | effective | / |
| *DPAGT1* | childhood | / | intellectual disability | normal | decrement | TA | effective | effective | ineffective |
| *ALG2* | infancy to childhood | / | no | normal;  slightly high | decrement | TA | effective or  ineffective | effective | ineffective |
| *ALG14* | infancy to childhood | / | epilepsy intellectual disability | normal | decrement | TA | effective | / | / |
| *GMPPB* | infancy to adulthood | LGMD | no | Very high | decrement | hypoglycosylation  of α-dystroglycan | effective | / | effective |
| *CHAT* | neonatal | hypoxic episodes | cerebral atrophy | normal | normal at rest; decrement with 10Hz RNS | NS | effective | / | effective |
| *COLQ* | neonatal or infancy | slow pupillary response scoliosis | no | normal | decrement repetitive CMAP | NS | ineffective or worsened | effective | / |
| *AGRN* | early childhood | Some predominantly  distal weakness | no | Normal;  slightly high | decrement some post exercise increment | NS | ineffective or  mildly effective | worsened | ineffective or slightly effective |
| *MUSK* | neonatal | some respiratory and bulbar involvement | no | normal | decrement | NS | ineffective or  worsened | effective | effective |
| *DOK7* | childhood | scoliosis | no | normal;  mildly high | decrement | NS | ineffective or  worsened | effective | effective |

TA, Tubular aggregates; LGMD, Limb Girdle Muscular Dystrophy; NS, Non-specific changes
